# Assessment of synthetic floral-based attractants and sugar baits to capture male and female *Aedes aegypti* (Diptera: Culicidae)

**DOI:** 10.1186/s13071-016-1946-y

**Published:** 2017-01-17

**Authors:** Kara Fikrig, Brian J. Johnson, Durland Fish, Scott A. Ritchie

**Affiliations:** 1 Yale School of Public Health, Yale University, 60 College Street, P.O. Box 208034, New Haven, CT 06520 USA; 2 College of Public Health, Medical and Veterinary Sciences, James Cook University, PO Box 6811, Cairns, QLD 4870 Australia; 3 Australian Institute of Tropical Health and Medicine, James Cook University, PO Box 6811, Cairns, QLD 4870 Australia

**Keywords:** *Aedes aegypti*, Entomological surveillance, Mosquito trap, Floral lures, Sugar lures, Zika, Dengue

## Abstract

**Background:**

The viruses transmitted by *Aedes aegypti,* including dengue and Zika viruses, are rapidly expanding in geographic range and as a threat to public health. In response, control programs are increasingly turning to the use of sterile insect techniques resulting in a need to trap male *Ae. aegypti* to monitor the efficacy of the intervention. However, there is a lack of effective and cheap methods for trapping males. Thus, we attempted to exploit the physiological need to obtain energy from sugar feeding in order to passively capture male and female *Ae. aegypti* (nulliparous and gravid) in free-flight attraction assays. Candidate lures included previously identified floral-based (phenylacetaldehyde, linalool oxide, phenylethyl alcohol, and acetophenone) attractants and an attractive toxic sugar bait-based (ATSB) solution of guava and mango nectars. A free-flight attraction assay assessed the number of mosquitoes attracted to each candidate lure displayed individually. Then, a choice test was performed between the best-performing lure and a water control displayed in Gravid *Aedes* Traps (GAT).

**Results:**

Results from the attraction assays indicated that the ATSB solution of guava and mango nectars was the most promising lure candidate for males; unlike the floral-based attractants tested, it performed significantly better than the water control. Nulliparous and gravid females demonstrated no preference among the lures and water controls indicating a lack of attraction to floral-based attractants and sugar baits in a larger setting. Although the guava-mango ATSB lure was moderately attractive to males when presented directly (i.e. no need to enter a trap or other confinement), it failed to attract significantly more male, nulliparous female, or gravid female *Ae. aegypti* than water controls when presented inside a Gravid *Aedes* Trap.

**Conclusions:**

Our findings suggest that the use of volatile floral-based attractants and sugar mixtures that have been identified in the literature is not an effective lure by which to kill *Ae. aegypti* at ATSB stations nor capture them in the GAT. Future trapping efforts would likely be more successful if focused on more promising methods for capturing male and female *Ae. aegypti*.

**Electronic supplementary material:**

The online version of this article (doi:10.1186/s13071-016-1946-y) contains supplementary material, which is available to authorized users.

## Background

The resurgence of once geographically limited vector-borne diseases, particularly those viruses transmitted by mosquitoes such as dengue, Zika and chikungunya viruses, have become an increasingly serious threat to public health in recent years. The expansion of these diseases is largely spurred by anthropogenic activities including increased mobility of human populations, habitat modification, and climate change [1, 2]. In the wake of these changes, vector-borne diseases are moving from one place to another at unprecedented rates, causing progressively more people to be at risk of contracting these diseases [1, 2]. In particular, diseases transmitted by *Aedes aegypti*, the yellow fever mosquito, have experienced a notable resurgence and expansion in recent years, especially Zika and dengue viruses [3, 4]. In the past year, Zika virus has spread through much of the Western Hemisphere, resulting in a public health emergency due to its likely association with microcephaly, a serious congenital malformation [5–7]. Furthermore, the incidence of dengue has increased 30-fold in the past 50 years. As a result, 2.5 billion people currently live at risk of contracting this disease, and there are 50 million infections and 22,000 deaths per year [3]. The cost of dengue on public health is substantial, including direct costs to local and global health organizations and immense economic and social costs [8, 9].

In response to the threat of dengue in endemic countries (and impending threat in non-endemic countries), scientists have increasingly turned to population level manipulations that rely upon males for optimal efficiency and successful dissemination. For example, the Eliminate Dengue program releases both male and female *Ae. aegypti* infected with *Wolbachia,* which reduces the ability of the mosquito to transmit dengue virus [10]. Additionally, biotech companies, such as Oxitec, produce genetically engineered sterile male *Ae. aegypti*, which suppresses vector population levels [11]. Other male-based sterile insect techniques (SITs), including radiation and feeding with double stranded RNA, also rely upon the release of sterile males for control of *Ae. aegypti* [12–14]*.* With the advent of such technologies, it has become increasingly important to trap males, in addition to females (the traditional target of mosquito control programs) in order to monitor the efficacy of these technologies. However, the tools available to sample wild male *Ae. aegypti* are limited. Those that do exist, such as the Biogents Sentinel Trap (BGS), are prohibitively expensive for large, wide-ranging studies and rely upon energy from batteries or mains power to function [15]. The passive trap options available for the capture of female *Ae. aegypti*, such as the Gravid *Aedes* Trap (GAT) and autocidal gravid ovitrap, are more practical and affordable [16–18]. These traps mimic the ecological drivers of oviposition site selection, such as dark color and odor of fermented plant material, in order to lure gravid females into the trap to lay eggs. Naturally, this technique is not effective for male *Ae. aegypti*, so passive trap designs must rely upon other physiological needs pertinent to male survival, such as sugar-feeding. Both male and female *Ae. aegypti* acquire energy in the form of carbohydrates from plants. This is the only source of food for males, as opposed to females, which primarily derive energy from blood meals [19].

*Anopheles* control programs have already successfully implemented strategies that exploit the sugar-feeding behavior of mosquitoes in the form of attractive toxic sugar baits (ATSB) [20–22]. Attractive toxic sugar baits strategies use floral-based attractants from a flower or fruit to attract the mosquitoes, include sugar to induce feeding, and an oral toxin to kill the mosquitoes. The technique has resulted in substantial reductions in the mosquito populations at the sites where it has been tested [20–23]. *Aedes albopictus* control programs have also had similar success with ATSB [24–26].

This same physiological need could be harnessed in order to trap *Ae. aegypti*. Several floral-based attractants, including acetophenone and phenylacetaldehyde, have been shown to be attractive to *Ae. aegypti* in small scale experiments [27, 28]. In the discussion of each of these papers, the authors highlighted the potential to use the promising floral-based compounds identified as attractants in traps [27, 28]. Additionally, ATSB of guava-mango nectars has been successful in reducing *Ae. albopictus* populations [25], which is an attraction that may extend to *Ae. aegypti* due to the biological and ecological similarities between *Ae. aegypti* and *Ae. albopictus* [29]. Thus, the objective of this study was to assess the attraction and potential to capture male and female (nulliparious and gravid) *Ae. aegypti* using promising floral-based attractants, ATSB solutions, and combinations of both. Candidate lures were assessed in free-flight tent trials to determine if preliminary reports from previously published small-scale trials were indicative of success in larger settings. If successful, using ATSBs to passively capture male and female *Ae. aegypti* would enable the creation of a practical and economically viable trap to monitor and aid control programs, especially SIT programs.

## Methods

### *Aedes aegypti* colony

Mosquitoes used in the studies were from a colony established from eggs collected in ovitraps in Cairns (QLD, Australia) and were periodically supplemented with wild collections to maintain genetic vigor. Mosquito larvae were reared on fish food powder (TetraMin Rich Mix, Tetra Melle, Germany). Adults were fed on a 50% honey solution and were blood-fed 3 times per week using human volunteers (Human ethics approval from James Cook University H3555). Mosquitoes between one and two weeks old were starved overnight prior to use in trials (about 18 h).

### Lure selection and presentation

#### General lure selection and presentation

Based on the literature and preliminary comparisons, five potential lures were chosen including three volatile floral-based attractants compound lures, one sugar lure and one combination combining floral attractants and sugar lure (Table 1). The three floral-based attractants were presented on a 3 × 3 cm sponge soaked in 12 ml of distilled water in volumes of 200 μl for phenylacetaldehyde, 200 μl for acetophenone, and a combination of 50 μl each of phenylacetaldehyde, linalool oxide, phenylethyl alcohol and acetophenone. An ATSB lure was created based on a recent recipe shown to be highly attractive to *Ae. albopictus* [25]. Initial sugar lure preparation involved the mixing of 0.2 l of guava nectar (Golden Circle), 0.2 l of mango nectar (Golden Circle), 0.2 l of distilled water and 200 g of brown sugar in an Erlenmeyer flask over heat using a heating pad and magnetic stirrer. When the sugar was fully suspended, the mixture was poured into a plastic container and allowed to cool and ferment for 24 h at room temperature. This mixture is referred to as guava-mango through the remainder of the paper. For each trial, 12 ml of guava-mango was pipetted onto a sponge for presentation. A combination lure of guava-mango and phenylacetaldehyde was also created by soaking the sponge with 12 ml of guava-mango and 200 μl of phenylacetaldehyde. All lures were displayed on a piece of sponge that was previously soaked in a 1% sodium hypochlorite (Chlorox®, Oakland, CA, USA) solution to dispel the chemicals used for packaging, after which the sponge was thoroughly rinsed and dried. The same procedure is used to sugar-feed the mosquitoes with 50% honey solution for lab-rearing.

**Table 1:** Synthetic floral-based attractants and sugar lures assessed in this study for the collection of *Ae. aegypti*

Treatment	Study design	Reference
Phenylacetaldehyde	electroantennography, wind tunnel bioassays	Jhumur et al. [28]
Phenylacetaldehyde + linalool oxide + phenylethyl alcohol + acetophenone	electroantennography, wind tunnel bioassays	Jhumur et al. [28]
Acetophenone	Y tube olfactometer	Von Oppen et al. [27]
Guava-mango	small screen cage studies, semi-field and field evaluations	Naranjo et al. [25]
Guava-mango + phenylacetaldehyde	semi-field cage evaluation	Fikrig et al. unpublished data

#### Development and validation of blue dye and fipronil to assess sugar-feeding

**Fig. 1:**
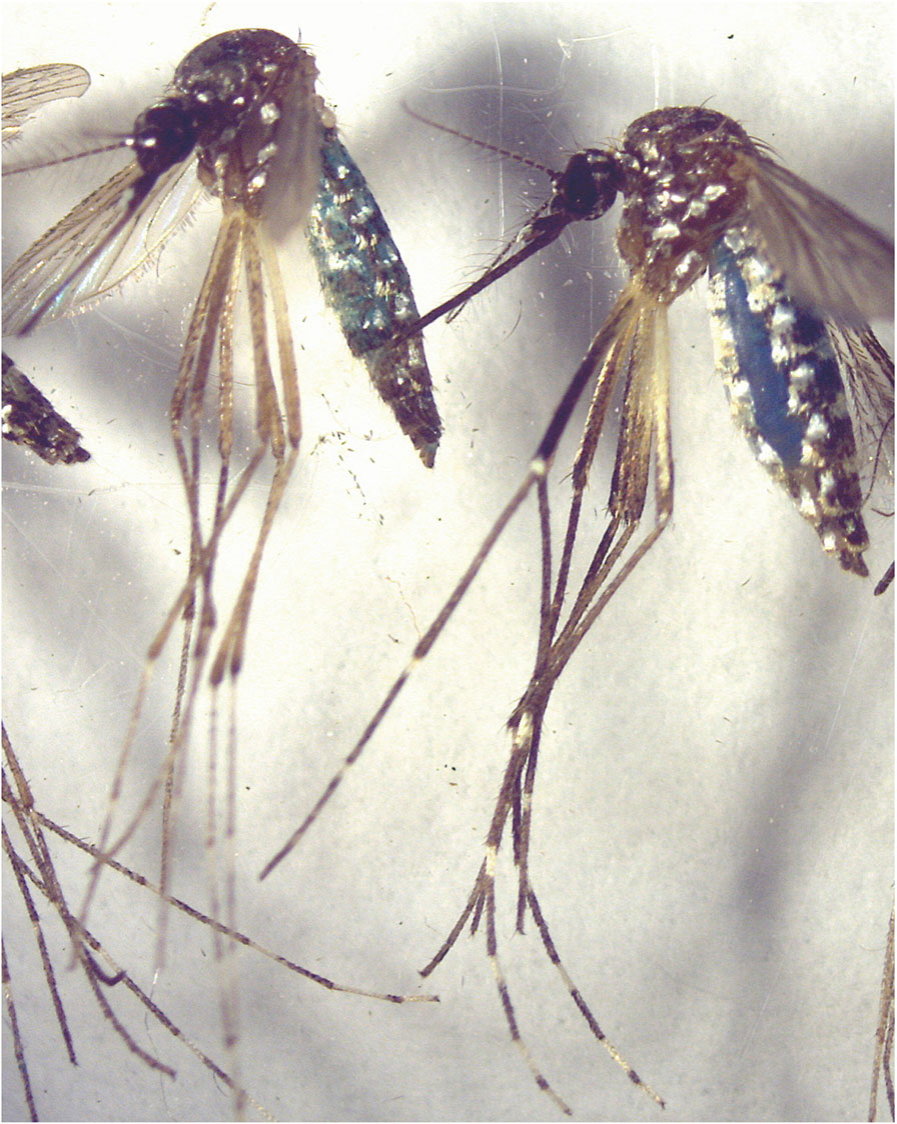
Example of blue honey solution visible in the abdomens of female *Aedes aegypti*

To assess sugar-feeding we incorporated blue food dye and the insecticide fipronil (0.06% by volume, Termidor®, Victoria, Australia) [30–32] in our control (distilled water) and treatment solutions (floral-based attractants and sugar lures) to knock down mosquitoes that ingested the lure and provide a visible blue dye in the abdomen (Fig. 1) to indicate that death was caused by ingestion and not natural causes. The rationale was that a larger number of *Ae. aegypti* dead after 24 h would mean that a larger number of mosquitoes ingested the lure from the sponge, which would suggest that the mosquitoes were more attracted to that lure.

The time to death or incapacitation after ingesting fipronil was determined by aspirating 13 male *Ae. aegypti* into a rearing cage (Bioquip Bug Dorms; 30 × 30 × 30 cm) with 12 ml of guava-mango treated with blue dye and fipronil and observing the number of landings on the sponge and the number of knock-down mosquitoes over two hours. We tested if mosquitoes readily fed on fipronil-treated lures by aspirating 20 male *Ae. aegypti* into two buckets with mesh covering. The treatment bucket contained a sponge with 12 ml of guava-mango treated with blue dye and fipronil while the control bucket contained a sponge with 12 ml of guava-mango treated only with blue dye. The number of mosquitoes in the treatment bucket that were killed and had visible blue in their abdomen was counted. The number of live mosquitoes in the control bucket was counted and then frozen so that the number with visible blue in the abdomen could be counted. The number that ingested the guava-mango was compared between treatment and control to see if there was any aversion to the fipronil treatment. The same procedure was repeated with 13 females.

### Are *Ae. aegypti* attracted to floral-based attractants and sugar lures?

For the attraction assay, we set up six tents (3.24 m^3^, Wild Country, Alfreton, United Kingdom) in a temperature and humidity controlled semi-field cage. The temperature and humidity in the semi-field cage track those of the outdoors, reflecting normal conditions in Cairns, Australia between June and August. The mean daily high temperature for June, July and August was 26.8 °C, 25.9 °C, and 27.1 °C, respectively, and the mean daily low temperature was 20.1 °C, 17.4 °C, and 16.6 °C, respectively [33]. The floors of the tents were covered with white tarp so that the dead mosquitoes would be easily spotted for counting. An overturned black plastic bucket from a Gravid *Aedes* Trap (GAT) [16] was placed at the middle of each tent to attract and induce swarming by male mosquitoes and provide a resting site for females. A small dish with the lure-treated sponges was placed on top of each GAT bottom. This feature was added after preliminary trials showed very little interaction with the sponge in the absence of a swarm marker or resting site.

We released 20 male *Ae. aegypti* into each tent after starving them overnight (about 18 h). They were left in the tent for 24 h, after which the dead mosquitoes on the ground of the tent were counted and inspected under a stereo microscope for blue dye in the abdomen or crop. The number with visible blue dye was noted, as this was considered the number definitively killed by the fipronil-treated attractant or control. The remaining live mosquitoes were cleaned from the tent using a Prokopak aspirator [34]. This procedure was repeated five times, for a total of five replications. The tent in which each of the five different treatments and the control were placed for each replication was randomized using the random number generator random.org. The same procedure was used with 20 nulliparous females as well as with 20 gravid female *Ae. aegypti.* The gravid females were blood fed six to seven days prior to use. Both the nulliparous and gravid females were starved overnight before use in the trials.

### Can the guava-mango lure be used to trap *Ae. aegypti* in the GAT?

#### Choice test between guava-mango lure and water control

**Fig. 2:**
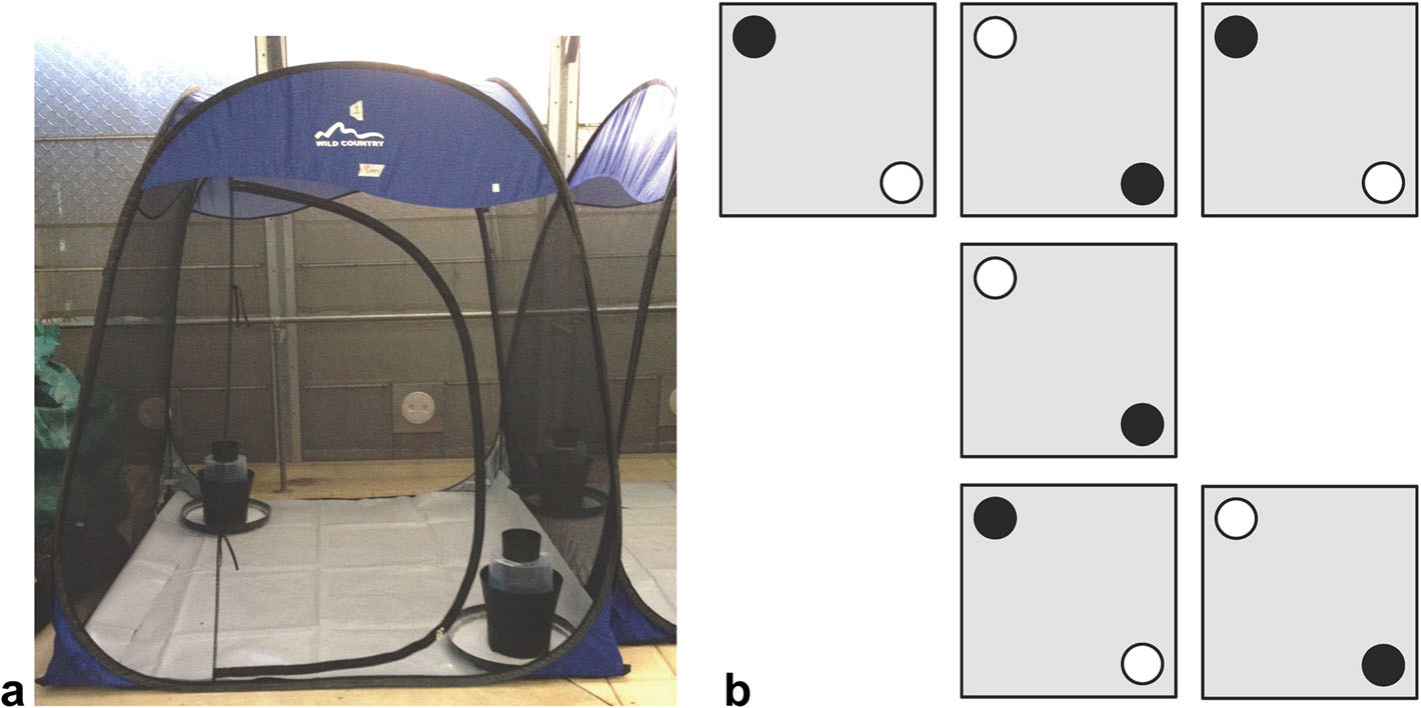
Image of tent and diagram of experimental setup. **a** Picture of tent with treatment and control GAT. **b** Diagram of experimental setup in the semi field cage. The grey squares represent the floor of the tents, the black circles represent the GATs treated with guava-mango and the white circles represent the control GATs

The attraction assays indicated that the ATSB guava-mango solution was the most attractive lure for male *Ae. aegypti,* so a secondary experiment was conducted to assess the potential to use this attraction to capture mosquitoes in the GAT. The guava-mango lure was prepared in the same way as in the attraction assay preparation, however the blue dye and fipronil were not added. The same six tents described for the attraction assay were used for the choice test. Each tent had two GATs set up at opposite corners across a diagonal (Fig. 2). The GATs were ~ 1/5 filled with tap water and five alfalfa pellets. Insecticide-treated bed net (5% alphacypermethrin) was placed over the screens of the GAT heads in order to knock down any mosquitoes that entered the traps. Each GAT was placed on a circular tray covered with talcum powder to prevent ants from entering the trap. In each tent, one GAT was the control, with a sponge soaked in 12 ml of distilled water in a plastic dish. The other GAT was the treatment, with a sponge soaked in 12 ml of the guava-mango lure in a plastic dish. In order to control for placement bias, the placement of the control and treatment GATs was switched in every other tent. Therefore, three of the tents had the guava-mango GAT in the far left corner and three had the guava-mango GAT in the near right corner, while the control GAT was at the other side of the diagonal in each tent. Twenty male *Ae. aegypti* were released into each tent and left for three nights (about 72 h). Thereafter, we removed the GATs from the tents and counted the number of mosquitoes in each. The same procedure was repeated with four tents over a separate 72 h for a total of 10 replicates. The procedure was also repeated with 20 nulliparous females and with 20 gravid female *Ae. aegypti.* Six replicates were conducted for each of these experiments using the six tents over one 72 h period in both cases.

#### Larger scale choice test

In a separate experiment, we increased the amount of liquid presented in each GAT. We scaled it up ten-fold from 12 ml to 120 ml to see if larger quantities would improve results. A full sponge was placed in a larger dish in the treatment and control GATs in order to absorb the increased quantity of guava-mango and water respectively. This was conducted in the six tents over a 72 h period for a total of six replicates. The same procedure was followed as described for the 12 ml paired test. However, this larger scale version was only conducted with male mosquitoes (20 male *Ae. aegypti* per tent).

### Statistical analysis

Analysis was conducted using SAS Studio 3.5. All data sets were tested for normality using the Shapiro-Wilk, test. Due to non-normality in all of the datasets, Kruskal-Wallis tests were used to compare the number of mosquitoes killed among the treatments and controls in the attraction assays. Due to non-normality in the male dataset, non-parametric Wilcoxon signed rank tests for paired samples were used to compare the number of mosquitoes captured by the sugar lure (guava-mango) and control GATs in the choice test.

## Results

### Validation of blue dye and fipronil to assess sugar-feeding

The observation of 13 male *Ae. aegypti* in a rearing cage with fipronil-treated guava-mango lure showed that those males that ingested the lure were knocked down within two hours of exposure. This shows the quick lethal action of the insecticide in *Ae. aegypti.* The comparison of 18 males fed with fipronil-treated guava-mango juice versus 19 males fed with insecticide-free guava-mango juice demonstrated that there is no aversion to fipronil. All 18 of the mosquitoes in the fipronil-treated bucket died, whereas none of the 17 mosquitoes in the untreated control died. Additionally, 15 of the 18 males in the fipronil-treated bucket had a visibly blue abdomen, which indicated substantial consumption of the guava-mango juice due to the blue food dye. In the untreated bucket, all 19 males had a visibly blue abdomen. The same comparison was repeated with 13 females in the bucket with fipronil-treated guava-mango juice and 13 females in the bucket with untreated guava-mango juice. The fipronil-treated bucket resulted in 13 dead mosquitoes, all with a blue abdomen. The untreated bucket resulted in 13 live mosquitoes, all with blue abdomen.

### Attraction assays

#### Male response to attraction assay

**Fig. 3:**
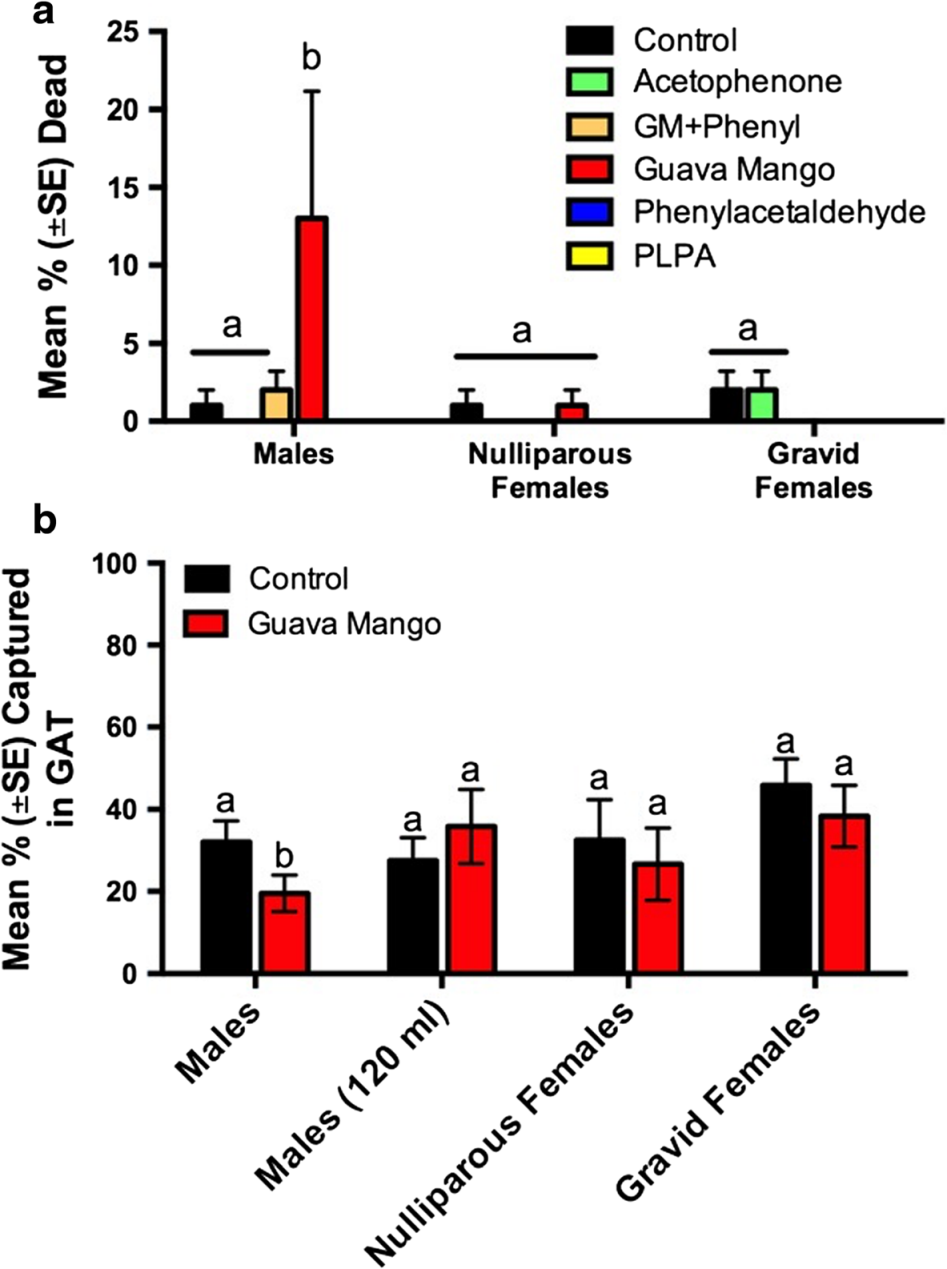
Percent of *Ae. aegypti* killed and captured in the attraction assay and choice test. **a** The percent of total number of mosquitoes released found dead with blue abdomens for each treatment in the attraction assays (*n* = 5). **b** The percent of total number of mosquitoes released caught in GATs baited with the guava-mango lure or water controls over a 72 h period. All experiments used 12 ml of guava-mango and control solution except one in which 120 ml of solution was used to assess the effect of dosage. For both experiments 20 mosquitoes were released during each replicate with a minimum of five replicates being completed for each of the lures and control. Different letters indicate significant differences among the treatments (*P* < 0.05)

To measure the efficacy of each lure, we measured the number of mosquitoes dead and the number of mosquitoes with blue coloration in the abdomen after 18 h (Fig. 3a, Table 2). The non-parametric Kruskal-Wallis test was significant (*χ* ^2^_(5,5)_ = 14.73, *P* = 0.01), indicating that one of the lures performed significantly differently from the other lures and the control. The guava-mango lure attracted and killed the highest mean number of male *Ae. aegypti* and performed particularly well in a couple of replicates (Additional file 1: Table S1), with 13.0 ± 8.2% of released males observed dead with indication of ingestion of the lure (Table 2, Fig. 3a).

**Table 2:** Attraction assay results. The mean percentage (± SE) of dead *Ae. aegypti* observed in the tent attraction assays with blue abdomens indicating ingestion of the lure. Twenty mosquitoes released for 18 h (*n* = 5). *P*-values determined following Kruskal-Wallis test

Treatment	Males		Nulliparous		Gravid	
	Mean %	*P =* 0.01	Mean %	*P =* 0.53	Mean %	*P =* 0.11
Control	1.0 (1.0)		1.0 (1.0)		0.4 (0.2)	
Acetophenone	0.0 (0.0)		0.0 (0.0)		0.4 (0.2)	
Guava-mango	13.0 (8.2)		1.0 (1.0)		0.0 (0.0)	
Phenylacetaldehyde	0.0 (0.0)		0.0 (0.0)		0.0 (0.0)	
Guava-mango + phenylacetaldehyde	2.0 (1.2)		0.0 (0.0)		0.0 (0.0)	
PLPA	0.0 (0.0)		0.0 (0.0)		0.0 (0.0)	

*Abbreviation: PLPA* phenylacetaldehyde + linalool oxide + phenylethyl alcohol + acetophenone

#### Female response to attraction assay

No significant difference in attractiveness was observed among the treatments and controls as indicated by the number of nulliparous (*χ* ^2^_(5,5)_ = 4.14, *P* = 0.53) and gravid (*χ* ^2^_(5,5)_ = 8.92, *P* = 0.11) females with blue-colored abdomens (Table 2, Fig. 3a).

### Choice test between guava-mango lure and water control

#### Male response

When compared directly to a water control, the guava-mango lure was not successful in attracting male *Ae. aegypti* into the GAT (Table 3, Fig. 3b). Overall, a mean (± SE) percentage of released male *Ae. aegypti* that entered and died in the GAT baited with guava-mango lure was 19.5 ± 4.5% after 10 replicates, whereas a mean of 32.0 ± 5.2% of released males entered and died in the GAT baited with water control (Fig. 3b). Analysis showed that the preference for the control over the guava-mango lure was significant (*Z =* 2.22, *P* = 0.03).

**Table 3:** Choice test results: The mean percentage (± SE) of dead *Ae. aegypti* observed in the guava-mango GAT choice test. Twenty mosquitoes released for 72 h. *P*-values determined from the Wilcoxon signed rank test

Group	N	Mean % in guava-mango	Mean % in control	*P-*value
Male	10	19.5 (4.5)	32.0 (5.2)	0.03
Male (120 ml)	6	35.8 (9.1)	27.5 (5.6)	0.69
Nulliparous	6	26.7 (8.8)	32.5 (9.9)	0.87
Gravid	6	38.4 (7.5)	45.9 (6.5)	0.47

#### Nulliparous females

The guava-mango lure was not successful in attracting nulliparous female *Ae. aegypti* into the GAT (Table 3, Fig. 3b). The mean (± SE) percentage of female *Ae. aegypti* that entered and died in the GAT baited with guava-mango or the control lure was 26.7 ± 8.8% and 32.5 ± 9.9%, respectively. There was no significant difference between the guava-mango and the control (*Z* = 0.16, *P* = 0.87).

#### Gravid females

The guava-mango lure was not successful in attracting gravid female *Ae. aegypti* into the GAT (Table 3, Fig. 3b). The mean percentage of gravid female *Ae. aegypti* that entered and died was 38.3 ± 7.5% in the GAT treated with guava-mango lure and 45.8 ± 6.5% in the control GAT. There was no significant difference between guava-mango and control (*Z* = 0.73, *P* = 0.47).

#### Larger scale lure

When the amount of guava-mango lure was increased by ten times the volume, it was still not successful in attracting male *Ae. aegypti* into the GAT (Table 3, Fig. 3b). The mean percentage of released male *Ae. aegypti* that entered and died was 35.8 ± 9.1% in the GAT treated with guava-mango lure and 27.5 ± 5.6% in the control GAT. Analysis showed that there was no significant difference between the capture of the guava-mango and control GATs (*Z* = -0.40, *P* = 0.69).

## Discussion

The floral-based attractants and ATSB lures reported as attractive to *Aedes* mosquitoes did not attract male *Ae. aegypti*, nor females regardless of physiological status (i.e. gravid or nulliparous) when presented in larger enclosures (tents) more reflective of field conditions*.* In the attraction assay, the guava-mango ATSB attracted significantly more males than the other four lures and the control, however the average percentage of mosquitoes killed was low (13 ± 8.2%), indicating that it would be an inefficient attractant. Nonetheless, the guava-mango lure performed particularly well in a couple of replicates, killing more mosquitoes than observed in any of the replicates of the other lures and control (Additional file 1: Tables S1, S2, S3). Since the main interest of the study was to find a lure that could successfully trap males for control and monitoring purposes, we proceeded with the most promising lure among the male trials. The next step was to bait a GAT with the guava-mango lure and pair it with a water control to compare the number of *Ae. aegypti* captured in each. The guava-mango GAT did not capture significantly more male, nulliparous or gravid female *Ae. aegypti* than the control GAT.

These results were unexpected because the bulk of the pre-existing literature suggested that male *Ae. aegypti* rely upon plant sugars to satisfy their energetic requirements [19]. Additionally, previous studies identified specific floral-based attractants that are particularly attractive to *Ae. aegypti* in small-scale enclosures and olfactometer studies under laboratory conditions. Furthermore, a sugar lure was identified as a successful attractant for *Ae. albopictus* in ATSB field studies [25]. The existing evidence therefore suggested that these floral-based attractants and sugar lures would be attractive to *Ae. aegypti* on a larger spatial scale and may facilitate passive trapping of males and females. However, once tested, this was not the case.

Despite the widespread acceptance that *Ae. aegypti,* especially males, derive energy from flower nectar [19], there is a growing body of evidence that the extent of this behavior is limited, especially among females [35, 36]. A mark-release-recapture study in Thailand showed that females in the field did not consume sugar over a two to three day period. In the same study, only one third of male *Ae. aegypti* consumed sugar [36]. Furthermore, there is evidence that suggests that in females, sugar feeding may be detrimental to survival when compared to blood feeding alone [37]. The conclusions reached in the aforementioned papers support the conclusion of our study, that the impetus to sugar feed is not strong enough to merit the basis of a lure for passive trapping. One of the reasons why *Ae. albopictus* and *Ae. aegypti* may differ in their readiness to sugar feed is how *Ae. aegypti* and *Ae. albopictus* interact with and rely upon vegetation. For example, *Ae. albopictus* tends to thrive in more highly vegetated habitats with a presumably high density of natural sugar sources, whereas *Ae. aegypti* prefers urbanized landscapes, often harboring inside houses and buildings, with presumably lower densities of natural sugar sources [38, 39]. These differences in habitat preference may have selected for a greater attraction to natural floral sugar sources in *Ae. albopictus* than *Ae. aegypti* and may account for the inconsistent attraction to the ATSB lure among *Ae. aegypti*.

Our findings suggest that it is inefficient to use these floral-based attractants and sugar lures in ATSB stations for the control of *Ae. aegypti* populations. The results also indicate that it is ineffective to use these lures to target sugar-feeding behavior in order to trap male and female *Ae. aegypti* passively*.* In contrast, recent research has shown that sound lures mimicking the tone of the female wing beat frequency provide a highly effective mechanism to trap male *Ae. aegypti* passively [40]. This may be a promising alternative to the lures investigated in this study for the capture of male *Ae. aegypti*. As for females, the use of simple gravid traps, such as the CDC Autocidal Gravid Trap and GAT [41, 42], and the use of BG-Sentinel traps (with and without CO_2_) are effective means of sampling gravid and nulliparious female *Ae. aegypti* and *Ae. albopictus* [43, 44].

## Conclusions

The attraction assay demonstrated that the floral-based attractants and sugar mixtures previously identified in the literature as potential trap lures were not more attractive than water to female (nulliparous and gravid) *Ae. aegypti*. The most promising of these lures for males, a combination of guava and mango nectars, did not facilitate passive trapping of the mosquitoes. These data suggest that the use of the volatile floral-based attractants and sugar mixtures that have been identified in the literature is not an effective lure to passively capture male or female *Ae. aegypti* in the GAT or at ATSB stations*.* Although our results do not support the use of the floral-based attractants and ATSB baits tested as trap lures, further work is needed that includes other characteristics of natural sugar sources (e.g. structural and color components of natural floral sugar sources) before their utility as attractants can be fully assessed. However, as it stands now, current efforts to trap male *Ae. aegypti* would likely have improved success if focused on more promising methods.

## Additional file

Additional file 1: Table S1. Summary of data collected in the male attraction assay. The information provided includes treatment type, replicate number, tent site (position in the flight cage), number of dead mosquitoes, number of dead mosquitoes with blue abdomens, and the date of data collection. **Table S2.** The same information was provided as in Additional file 1: Table S1 for the data collected in the nulliparous female attraction assay. **Table S3.** The same information was provided as in Additional file 1: Table S1 for the data collected in the gravid female attraction assay. **Table S4.** Summary of data collected in the male (12 ml) choice test, including the number of males collected in the guava-mango and control GATs. **Table S5.** Summary of data collected in the nulliparous female choice test, including the number of nulliparous females collected in the guava-mango and control GATs. **Table S6.** Summary of data collected in the gravid female choice test, including the number of gravid females collected in the guava-mango and control GATs. **Table S7.** Summary of data collected in the male (120 ml) choice test, including the number of males collected in the guava-mango and control GATs. (XLSX 46 kb)

## Abbreviations

ATSB: Attractive toxic sugar bait
BGS: Biogent sentinel trap
GAT: Gravid *Aedes* trap
GM: Guava-mango
Phenyl: Phenylacetaldehyde
PLPA: Phenylacetaldehyde + linalool oxide + phenylethyl alcohol + acetophenone
SIT: Sterile insect technique
